# An update of posterior retroperitoneoscopic adrenalectomy – Case series

**DOI:** 10.1016/j.ijscr.2020.04.101

**Published:** 2020-05-16

**Authors:** Carlos E. Costa Almeida, Teresa Caroço, Marta A. Silva, José M. Baião, Ana Costa, Miguel N. Albano, João M. Louro, Luis F. Carvalho

**Affiliations:** a General Surgery, Centro Hospitalar e Universitário de Coimbra (Hospital Geral - Covões), Quinta dos vales, São Martinho do Bispo, 3041-853 Coimbra, Portugal; b General Surgery, Instituto Português de Oncologia de Coimbra Francisco Gentil, Av. Bissaya Barreto, 2005 Coimbra, Portugal

**Keywords:** Adrenal, Posterior retroperitoneoscopic adrenalectomy, Minimal invasive surgery, Endocrine

## Abstract

**Introduction:**

Posterior retroperitoneoscopic adrenalectomy (PRA) has advantages over transperitoneal approach. A second group of 10 patients is analyzed and compared with the first 10 procedures. Conclusions on feasibility, safety and learning curve are taken.

**Material and methods:**

A retrospective analysis of a second group of 10 patients submitted to PRA was conducted. All patients with functioning and non-functioning adrenal tumors <6–8 cm and without features of malignancy were included. A comparison with the previous 10 cases was conducted, and the results of all 20 cases were compared with other surgeons.

**Results:**

Pre-operative diagnoses: Conn’s syndrome – 8 (80%); Pheochromocytoma – 1 (10%); Non-functioning tumor (≥ 4 cm) – 1 (10%). Mean size of adrenal tumors was 2,9 cm. Mean operative time for first group was 46,7 min and 31,1 min for the second (p = 0,036). Postoperative in-hospital days decreased in the second group (p = 0,01). Conversion rate was equal (10%). Morbidity and mortality were similar.

**Discussion:**

Comparing the evolution of operative time in both groups, a constant and faster operative time was noted for the second group and a decreasing linear tendency was noted as more cases were being performed. Postoperative in-hospital days lowered in the second group, because with experience we started discharging patients earlier. Outcomes are stable between both groups. Our results match other authors data.

**Conclusion:**

These results are consistent with our first report and support the small learning curve for PRA, which is technically feasible and safe. Operative time and in-hospital days are influenced by surgeon’s experience. More cases need to be collected so that these results can be validated.

## Highlights

• Posterior retroperitoneoscopic adrenalectomy (PRA) has advantages over transperitoneal approach.
• Operative time and in-hospital days are influenced by surgeon’s experience.
• Conversion rate, morbidity and mortality are stable with experience.
• A fast-operative time may justify the low morbidity.
• PRA has a small learning curve, is feasible and safe.

## 1 Introduction

Posterior retroperitoneoscopic adrenalectomy (PRA) is an alternative to the laparoscopic adrenalectomy (LA), which is the gold standard since its description in 1992 by Michael Gagner [1]. However, PRA has some advantages over the transperitoneal approach (TA), namely a fast and direct access to the gland, no incursion into intraperitoneal space avoiding incidental trauma to viscera, reduced operative time, less pain and faster recovery [2,3]. Although its advantages, PRA is not the most chosen approach for adrenal masses because many surgeons are not familiarized with the retroperitoneal space, and they get the usual wider space with the transperitoneal technique [[3], [4], [5]]. The authors are using the PRA to treat adrenal tumors up to 6–8 cm without features of malignancy. The technique was learned in April 2014 from Prof. Dr. Martin Walz from Essen, Germany, and one year later we started using it in a routine basis. In 2018 we published the data from the first 10 cases operated between 2015 and 2018. Because our results were similar to more experienced surgeons, we could conclude that PRA is feasible, safe, and with a small learning curve [5]. The objective of this update is to analyze the second group of 10 cases operated between 2018 and 2019, compare with the results of the first 10 cases, to present the results of all 20 cases and conclude about the feasibility, safety and learning curve.

## 2 Material and methods

A retrospective analysis of the second group of 10 patients submitted to PRA was conducted. All patients were operated by the same surgeon (first author) who also operated the first 10 cases included in the first publication [5]. The author learned the technique from Prof. Dr. Martin Walz, who is responsible for the spread of this technique, and has experience in other fields of minimal invasive surgery. All patients with functioning and non-functioning adrenal tumors with <6–8 cm and without features of malignancy were included. Patients with concomitant abdominal pathologies needing treatment and patients with BMI > 35 were excluded. This is a case series study of consecutive patients treated in a single center (Academic Hospital) between January 2018 and December 2019. Data collected were the same as the first report: gender, age, pre-operative diagnosis, tumor size, side of tumor, operative time, conversion, postoperative in-hospital length of stay, morbidity and mortality. A minimum follow-up of three months was conducted for each and every patient. A comparison with the previous 10 cases was conducted, and the results of all 20 cases were compared with other surgeons.

A statistical analysis was conducted using the *t* test, comparing both groups according to size, operative time and post-operative days. Mean values are presented. A p < 0,05 was considered statistically significant. Because of the small number of patients this is a preliminary analysis.

No ethical approval was needed because posterior retroperitoneoscopic adrenalectomy is a well-known procedure and a valid option to treat adrenal masses. This study has the research registry number 5443. Written informed consent was obtained from all patients for publication of this case series. This paper has been reported in line with the PROCESS criteria [6].

## 3 Results

Between January 2018 and December 2019, a second group of ten (10) patients were submitted to posterior retroperitoneoscopic adrenalectomy (PRA) in our department. All patients were operated by the same surgeon, who has experience in other fields of minimally invasive surgery, namely colorectal, hepatobiliary and hernias.

The characteristics of the included patients are described in Table 1. Pre-operative diagnoses were as follows: Conn’s syndrome – 8 (80%); Pheochromocytoma – 1 (10%); Non-functioning tumor (≥4 cm) – 1 (10%). Mean size of adrenal tumors in this case series was 2,9 cm. The largest lesion was a non-functioning cyst with 11 cm.

**Table 1:** Data of all 20 patients. Partial means of both groups and global means for age, size, operation time and postoperative in-hospital days are presented.

		Gender	Age	Diagnosis	Size (cm)	Side	Conversion	Duration (min)	Post-op days	Complications	Mortality
**First 10 cases**											
**Patient**	**1**	M	63	Conn	2,0	right	no	40	2	no	no
	**2**	F	53	Non-fun	4,0	right	no	30	2	no	no
	**3**	M	54	Cushing	1,8	left	no	75	2	no	no
	**4**	M	52	Pheo	14,0	right	no	70	4	no	no
	**5**	F	63	Conn	1,8	left	no	30	2	no	no
	**6**	F	70	Pheo	3,0	left	no	45	4	no	no
	**7**	F	38	Cushing	2,8	left	no	30	2	no	no
	**8**	M	60	Pheo	5,0	left	yes	–	–	no	no
	**9**	F	43	Conn	2,0	left	no	30	1	no	no
	**10**	F	50	Conn	4,3	right	no	70	1	no	no
**Partial Mean**			**54,6**		**4,1**			**46,7**	**2,2**		
**Second 10 cases**											
**Patients**	**11**	F	62	Conn	2,0	left	no	25	1	no	no
	**12**	M	44	Conn	0,9	left	no	35	1	no	no
	**13**	M	62	Conn	1,7	right	no	40	1	no	no
	**14**	M	31	Non-fun	11,0	right	yes	–	–	no	no
	**15**	M	66	Conn	1,4	left	no	35	1	no	no
	**16**	M	52	Pheo	5,3	left	no	35	2	no	no
	**17**	F	43	Conn	1,6	right	no	30	1	no	no
	**18**	M	47	Conn	1,7	right	no	30	1	no	no
	**19**	F	43	Conn	1,4	left	no	20	1	no	no
	**20**	M	42	Conn	1,9	right	no	30	1	no	no
**Partial Mean**			**49,2**		**2,9**			**31,1**	**1,1**		
**Global Mean**			**51,9**		**3,5**			**38,9**	**1,7**		

Surgery was performed exactly as in the first ten cases. Three trocars were used: 12 mm balloon trocar (tip of the 12th rib), 5 mm (tip of the 11th rib) and 10 mm (midline between 12 mm trocar and spine). A 30° camera was used. A 5 mm laparoscopic LigaSure and a non-traumatic grasper were the only instruments used. Gland and tumor were retrieved in an endobag. Operative field was washed at the end with saline solution. No drainage. The fascial plane was closed with absorbable sutures, and skin was sutured with a non-absorbable suture.

Nine (9) patients were submitted to complete PRA, and only one patient (10%) had to be converted. This patient was the one with the giant non-functioning adrenal cyst who had a small iatrogenic inferior vena cava (IVC) injury. A lumbotomy was immediately performed (keeping the patient in prone position) and IVC was repaired with a non-absorbable suture and protected with a TachoSil® sponge. Operative time for complete PRA was between 20 and 40 min, with a mean operative time of 31,1 min. For this case series a mean of 1,1 days was found for post-operative in-hospital days. Blood loss was negligible (<20 mL). There were neither post-operative complications (0%) nor mortality cases (0%). Table 1. At three months of follow-up only the patient who was submitted to the lumbotomy was complaining of paresthesia in the right flank. All patients submitted to complete PRA had no complaints.

Table 1 presents the results of the first 10 cases followed by the second 10 cases submitted to PRA. Conversion rate was the same in both groups (10%), as well as morbidity and mortality rates (0%). Both groups were similar according to lesion size with a p = 0,447 (95% confidence interval: −2,009 to 4,369). Our mean operative time in the first 10 cases was 46,7 min (30 min–70 min), while in the second 10 cases a mean of 31,1 min was found. This difference is statistically significant with a p = 0,036 (95% confidence interval: 1,12 to 29,99). Post-operative days were also different in the second group. While in the first 10 cases a mean of 2,2 days was reported, in the second 10 cases a mean of 1,1 days was found. With a p = 0,01 (95% confidence interval: 0,30 to 1,92) there is also a statistical significance in post-operative in-hospital days. Table 2.

**Table 2:** Comparison between first 10 cases and second 10 cases. Mean values for all cases are also presented. Groups are similar according to tumor size (p = 0,447). The second group has a lower mean operative time (p = 0,036). Postoperative in-hospital days decrease in the second 10 cases (p = 0,01). Global mean operative time: 38,9 min.

	First 10 cases	Second 10 cases	All cases	p value
**Age**	54,6	49,2	**51,9**	
**Size**	4,1	2,9	**3,5**	**p = 0,447**
**Duration (min)**	46,7	31,1	**38,9**	**p = 0,036**
**Post-op days**	2,2	1,1	**1,7**	**p = 0,01**
**Conversation (%)**	10	10	**10**	
**Morbidity (%)**	0	0	**0**	
**Mortality (%)**	0	0	**0**	

## 4 Discussion

Posterior retroperitoneoscopic adrenalectomy (PRA) has advantages over transperitoneal approach (TA). The former allows for a fast and direct access to the adrenal gland without incursion into the peritoneal space, eliminating the risk for incidental trauma to abdominal viscera of the latter one and avoiding adhesions from previous surgeries [[3], [4], [5],^7^]. PRA has a faster recovery with less post-operative pain, has a shorter operative time and is feasible in obese patients [3,5]. Additionally, PRA is a safe technique for high risk patients (American Society of Anesthesiologists (ASA) score ≥ 3, anticoagulant therapy, BMI > 30) [8]. Even though, because surgeons are not familiarized with the retroperitoneal space and the transperitoneal approach offers the usual wider space of peritoneal cavity, PRA is still not the preferred technique [[3], [4], [5]]. Another issue is the lack of anatomical landmarks at the very beginning of the procedure. However, following important tips from experienced surgeons, even those who had never performed this technique but have minimally invasive surgery experience will be able to do it [5]. First, it is easy to enter the retroperitoneal space with a scissors. Second, place the lateral trocar with digital control. Third, open the Gerota’s fascia and lower all the fatty tissue before placing the medial trocar. Fourth, first step is to find an anatomical landmark, which is the upper pole of the kidney, by cutting through the Gerota’s fat.

After performing the first 10 cases of PRA, the authors found their results were similar to more experienced surgeons in terms of operative time, post-operative in-hospital days, morbidity and mortality. These results led to the conclusion that PRA is feasible, safe, and with a small learning curve if the surgeon has already gained experience in other fields of minimally invasive surgery [5]. After performing 10 more cases, the authors decided to compare both groups (first 10 cases vs second 10 cases). The questions are: Are results similar or is there any difference? Does more experience have any impact in the outcome?

Mean operative time of 46,7 min and 31,1 min were noted for first 10 cases and second 10 cases respectively (p = 0,036). By analyzing and comparing the evolution of operative time in both groups it seems clear that in the first group there was a wide variation (30–70 min), while in the second group a more constant and faster operative time was noted (25–40 min). Graphic 1. Additionally, when operative time of all cases is analyzed together, a decreasing linear tendency is noted as more cases are being performed, apparently towards a stable operative time around 30 min. Graphic 2. The authors hypothesized that differences in tumor size could influence these results. However, both groups were similar according to tumor size (p = 0,447). Table 2. Mean post-operative days reported for the first group was 2,2 days, contrasting to the 1,1 days for the second group (p = 0,01). This difference could be explained by the fact of the second group has less cases of pheochromocytoma who needed more post-operative in-hospital days (including one day in the Intensive Care Unit). Even though, there is only one more pheochromocytoma case in the first group, and the only patient with a pheochromocytoma in the second group was discharged home in the second post-operative day, two days earlier than in the first group. Table 1. This leads to the second explanation. With experience the authors started discharging patients earlier. This appears to be a more valid justification since in the second group patients with non-pheochromocytoma tumors were discharged home in the day after surgery, contrasting with the usual two days in the first group.

**Graphic 1:**
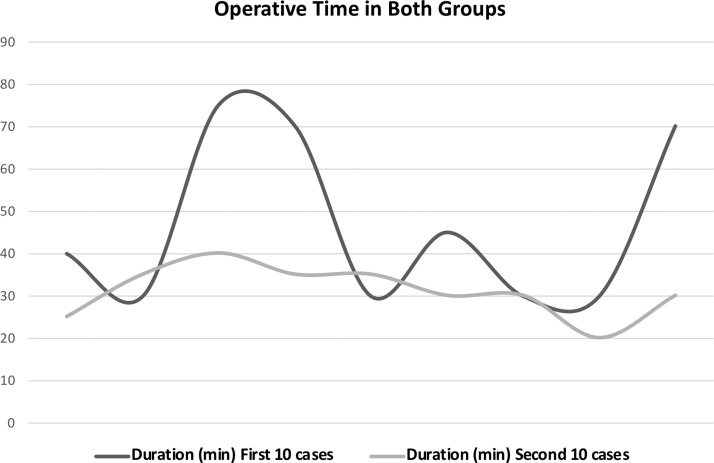
Evolution of operative time in both groups. In the first 10 cases operative time has a wide variation (30–70 min), while in the second 10 cases operative time is stable (20–40 min). p = 0,036.

**Graphic 2:**
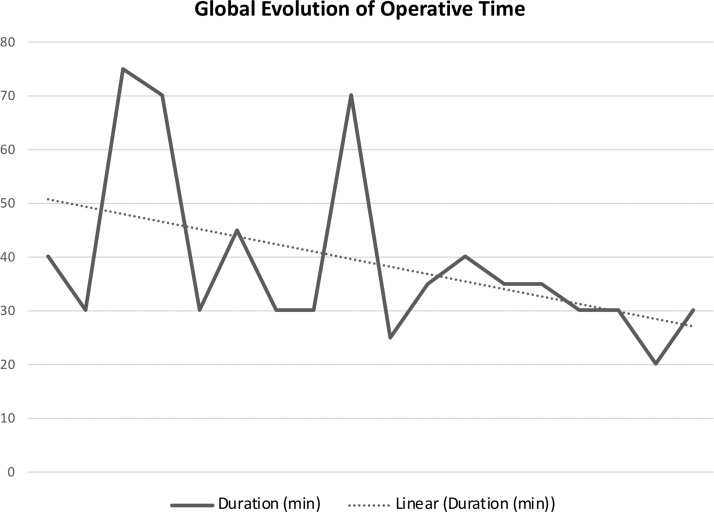
Global evolution of operative time. Operative time decreases as more procedures are performed. There is a linear descendent tendency, apparently towards a 30 min operative time.

Conversion rate of 10% was found in both groups. No post-operative complications nor mortality cases in the two groups were found. These results show there were no differences in the outcomes between the two groups. Only operative time (p = 0,036) and in-hospital days (p = 0,01) were influenced by surgeon’s experience. These data support the idea of a small learning curve for PRA. However, there is a major drawback of this study. The small number of patients in both groups does not allow to take valid conclusions. Eventually, as more and more cases are being collected, a comparison study between bigger groups will validate these results.

Since the first procedure, the authors are trying to include patients with bigger lesions. This is why a patient with an 11 cm cyst was treated by PRA. However, the outcome was not the expected. After puncture and aspirating the cyst, dissection was going well. With 90% of the cyst and gland freed, a small iatrogenic injury to the inferior vena cava (IVC) occurred. A lumbotomy was necessary for IVC repair. In the first group a patient with a giant cystic pheochromocytoma was included, and an incidental rupture of the lesion occurred [5,9]. Although there are no studies comparing PRA with TA and open surgery for bigger lesions, based in our experience we cannot advise the use of PRA for lesions ≥ 6–8 cm.

The authors also compared the results of all 20 patients submitted to PRA with results reported by experienced surgeons. Table 3. Walz el al. in a study of 560 procedures in 520 patients report a mean operative time of 67 ± 40 min, declining from the first cases (106 ± 46 min) to the later ones (40 ± 15 min) [7]. A paper from Cabalag et al. published in 2014 report a mean operative time of 70,5 min after the first 50 cases of PRA [10]. Porpiglia et al. from Italy report a mean operative time of 90 min (45–210 min) for 50 procedures [11]. Vrielink et al. report a global mean operative time of 89 min (29–265 min) for a total of 181 procedures, however the study included four different teams from different centers and it does not provide data for each one, which is why we did not include this study in Table 3 [3]. Lee and Kiriakopolous report a mean operative time of 105,6 min and 87,2 min, respectively [4,13]. For the first 10 procedures our mean operative time was 46,7 min, decreasing to 31,1 min for the following 10 procedures (p = 0,036). These results are better than other reports: 100 min for the first 20 procedures, declining to 83 min for the following 20 procedures [3]. Our global mean operative time for complete PRA is 38,9 min, with a descendent linear tendency towards a stable operative time around 30 min. We have a lower operative time than some reports [3,4,8,10,13], but consistent with experienced surgeons [7].

**Table 3:** Data published in worldwide literature (mean values). Our mean operation time is similar to Walz, but inferior to the majority of other authors. No morbidity or mortality. Conversion rate was higher than other studies. Blood loss and mean in-hospital days are similar to other authors. (N/A – not available).

	Walz et al. [7]	Cabalag et al. [8]	Porpiglia et al. [9]	Kiriakopoulos et al. [12]	Lee et al. [4]	CE Costa Almeida et al.
**N**	560	50	50	19	17	**20**
**Size (cm)**	2,9	N/A	N/A	3,7	2,64	**3,5**
**Operation time (min)**	40	70,5	90	105,6	87,2	**38,9**
**Blood loss (ml)**	10	N/A	50	N/A	20	**<20**
**Conversion (%)**	2	0	8	0	0	**10**
**In-hospital days**	N/A	1	N/A	2,1	3	**1,7**
**Morbidity (%)**	14,4	8	12,5	N/A	0	**0**
**Mortality (%)**	0	0	0	0	0	**0**

Worldwide literature reports a complication rate of 8–17%. Postoperative complications include pleural tear, pneumothorax, pneumonia, surgical site infection (SSI), bleeding, hypoesthesia and/or relaxation of abdominal wall [4,5,7,[10], [11], [12]]. A multicenter study from Vrielink et al. reports bleeding from a polar artery, bleeding from the 12th rib intercostal artery and splenic laceration treated by splenectomy as possible perioperative complications [3]. By not opening the peritoneal cavity PRA decreases post-operative ileus, avoids bowel adhesions from previous operations and avoids incidental trauma to abdominal viscera [3,5]. We report a postoperative complication rate of 0% for all cases submitted to complete PRA, which is below the majority of reports. Table 3. According to Christakis et al. a greater operative time and a smaller gland are linked to a higher complications rate [12]. So, having a low operative time can justify the low post-operative complications we have. Although a fast surgery is better for the patient, we believe it does not justify trying to operate faster and faster. Every surgeon has their pace and the final outcome is what dictates if a procedure is being well performed.

Conversion to laparotomy varies throughout worldwide literature. A mean conversion rate of 2% and 8% is reported by Walz and Porpiglia respectively, while Cabalag report a 0% rate [7,10,11]. Both Lee and Kiriakopoulos report 0% of conversions [4,12]. A multicenter study reports a conversion rate of 5%, but one of the four surgical teams included had none conversion out of 31 procedures [3]. Our conversion rate is stable in the 10% (2 patients), but even so is higher than other reports. This may be explained by the small number of cases, which is a major drawback of this study. Another reason is the fact that one conversion case was due to previous dorsal and lumbar trauma with scar tissue formation, which precluded working in the retroperitoneal space. The second case of conversion occurred after an iatrogenic injury to the IVC. The “sword fighting” caused by posterior retroperitoneoscopic approach precluded the repair, obligating the conversion to lumbotomy. This “sword fighting” must be taken into account when dealing with vascular lesions during PRA. It is always better to immediately convert to open surgery in order to promote a fast repair.

There are several reports demonstrating advantages of PRA over TA in terms of in-hospital days, minimal blood loss, reduced pain and faster recovery [[3], [4], [5],^10^]. Lee et al. report a mean in-hospital length of stay of 3 days and 5,92 days for PRA and TA respectively (p = 0,003) [4]. A comparison study between posterior retroperitoneoscopy and laparoscopy conducted by Kiriakopoulos present a mean hospital stay of 2 days for PRA and 40 days for TA [13]. Cabalag et al. report a mean post-operative length of stay of 1 day [10], while Lee report a mean of 3 days [4]. Blood loss was kept minimal and below 20 mL, matching other studies [4,7,12]. We found a global mean postoperative in-hospital days of 1,7. Table 3. This represents a huge advantage of this technique. It is interesting to note that in the second 10 cases there was a decrease in the in-hospital length of stay (p = 0,01), which is probably due to the increasing confidence of the surgeon as more experience is gained.

All the data presented above support the idea of our first report that PRA has a small learning curve and contradicts the idea of Vrielink et al. that 24–42 procedures are necessary to complete the learning curve [3].

## 5 Conclusion

PRA is feasible and safe. With a similar operative time to experienced surgeons and with no morbidity nor mortality, we conclude that these results are consistent with our first report and support the small learning curve for PRA. Operative time and in-hospital days seems to be influenced by surgeon’s experience. More cases need to be collected so that these results can be validated.

## Declaration of Competing Interest

All authors declare no conflicts of interest.

## Sources of funding

No funding for our research.

## Ethical approval

No ethical approval was needed because posterior retroperitoneoscopic adrenalectomy is a well-known procedure and a valid option to treat adrenal masses.

## Consent

Written informed consent was obtained from all patients for publication of this case series and accompanying images. A copy of the written consent is available for review by the Editor-in-Chief of this journal on request.

## Author contribution

CECA: study design, data collection, data analysis, writing the paper, review.

TC: data analysis, writing paper, review.

MS: data collection, review.

JMB: review.

AC: data collection, review.

MA: data collection, review.

JL: review.

LC: data collection, review.

## Registration of research studies

Name of the registry: An update of posterior retroperitoneoscopic adrenalectomy – case series.

Unique identifying number or registration ID: researchregistry5443.

Hyperlink to your specific registration (must be publicly accessible and will be checked): https://www.researchregistry.com/browse-the-registry#home/registrationdetails/5e779e426b71e100155de264/.

## Guarantor

Carlos M Costa Almeida.

## Provenance and peer review

Not commissioned, externally peer-reviewed.

## CRediT authorship contribution statement

**Carlos E. Costa Almeida:** Conceptualization, Methodology, Formal analysis, Investigation, Data curation, Writing - review & editing, Visualization, Project administration. **Teresa Caroço:** Formal analysis, Data curation, Writing - review & editing. **Marta A. Silva:** Writing - review & editing. **José M. Baião:** Writing - review & editing. **Ana Costa:** Writing - review & editing. **Miguel N. Albano:** Writing - review & editing. **João M. Louro:** Writing - review & editing. **Luis F. Carvalho:** Writing - review & editing.
